# Accumulation and Elimination Dynamics of the Hydroxybenzoate Saxitoxin Analogues in Mussels *Mytilus galloprovincialis* Exposed to the Toxic Marine Dinoflagellate *Gymnodinium catenatum*

**DOI:** 10.3390/toxins10110428

**Published:** 2018-10-26

**Authors:** Pedro Reis Costa, Ana Catarina Braga, Andrew D. Turner

**Affiliations:** 1 IPMA—Portuguese Institute for the Sea and Atmosphere, Av. Brasília, 1449-006 Lisbon, Portugal; ana.braga@ipma.pt; 2 CCMAR—Centre of Marine Sciences, University of Algarve, Campus of Gambelas, 8005-139 Faro, Portugal; 3 Biology Department and CESAM, Aveiro University, 3810-193 Aveiro, Portugal; 4 Centre for Environment Fisheries and Aquaculture Science (CEFAS), Barrack Road, Weymouth, Dorset DT4 8UB, UK; andrew.turner@cefas.co.uk

**Keywords:** saxitoxin, shellfish metabolism, paralytic shellfish poisoning, harmful algal blooms

## Abstract

Paralytic shellfish poisoning (PSP) is a severe food-borne illness, caused by the ingestion of seafood containing paralytic shellfish toxins (PST), which are naturally produced by marine dinoflagellates and accumulate in shellfish during algae blooms. Novel PST, designated as hydroxybenzoate analogues (also known as GC toxins), was relatively recently discovered in *Gymnodinium catenatum* strains worldwide. However, to date, there have been no studies examining their accumulation in shellfish. In this study, mussels (*Mytilus galloprovincialis*) were exposed to *G. catenatum* for five days and then exposed to a non-toxic diet for 24 h, to investigate the toxin’s accumulation/elimination dynamics. As determined by UHPLC-HILIC-MS/MS, the hydroxybenzoate analogues, GC1 to GC6, comprised 41% of the algae toxin profile and only 9% in mussels. Elimination of GC toxins after 24 h was not evident. This study highlights that a relevant fraction of PST in mussels are not routinely analysed in monitoring programs and that there is a need to better understand the toxicological potential of the hydroxybenzoate analogues, in order to properly address the risk of *G. catenatum* blooms.

## 1. Introduction

**Figure 1:**
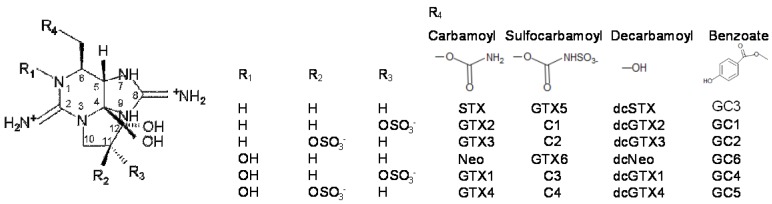
Chemical structure of the four groups of paralytic shellfish toxins (PST).

Paralytic shellfish toxins (PST) are potent neurotoxic alkaloids, produced in the marine environment by dinoflagellate species belonging to three genera, namely *Alexandrium*, *Pyrodinium*, and *Gymnodinium*. The toxicity of PST is caused by a high affinity inhibition of voltage-gated sodium channels (Na_v_) on the extracellular membranes of nerve cell terminals [1,2]. Structural differences among PST analogues—which were classically divided in three groups, the carbamoyl, dicarbamoyl, and sulfocarbamoyl groups—result in different affinities to the binding sites of Na_v_, leading to a varying degree of toxicity. A fourth group of PST was described in the early 2000s, in *Gymnodinium cateantum* strains from Australia, Uruguay, China, Spain, and Portugal [3]. This fourth group has the carbamate side chain replaced with a hydroxybenzoate moiety, as shown in Figure 1. The new toxins were designated as GC toxins (GC1–3), with GC3 corresponding to the 4-hydroxybenzoate ester derivative of decarbamoylsaxitoxin (dcSTX), and GC1 and GC2 to the epimeric sulphate derivatives of GC3 [3]. Indeed, *Gymnodinium catenatum* has been shown to produce a wide array of PST. In subsequent years, several other hydroxybenzoate analogues have been reported and designated as *p*-hydroxybenzoyl, di-hydroxybenzoyl, and sulfo-benzoyl derivatives [4,5,6,7]. The toxicity of GC toxins is still poorly known. In vitro studies, using rat brain synaptosomes with enriched sodium channels, indicate high affinity binding to Na_v_ [8], and computer simulations that modelled toxicity processes also suggest high affinity to Na_v_ [9].

Accumulation and transfer of PST throughout the marine food web may cause the death of marine wildlife and human intoxication [10,11]. Paralytic shellfish poisoning (PSP) is an illness characterized by neurological issues, from numbness of the fingers and extremities, tingling, nausea, and vomiting, to muscular paralysis and death by respiratory paralysis and cardiovascular shock [12,13]. In order to protect public health from acute intoxication, most coastal countries have a monitoring program in place that closes shellfish harvesting, whenever PSP toxicity exceeds 800 µg STX (saxitoxin) equiv.kg^−1^ in shellfish meat. From 1 January 2019, the EU reference method for determination of PSP toxins—based on pre-column oxidation (pre-cox) high performance liquid-chromatography with fluorescence detection (HPLC-FLD)—will come into effect. This method was developed to quantify “classic” hydrophilic carbamoyl, dicarbamoyl, and sulfocarbamoyl PST, but not the GC toxins. This may justify why the presence and variability of hydroxybenzoate saxitoxin analogues in shellfish have been poorly investigated. One attempt to quantify GC toxins in shellfish after a *G. catenatum* bloom was carried out by [4], using a modified and automated version of the HPLC-FLD method. Low levels of GC toxins were determined, suggesting their conversion into decarbamoyl toxins [4]. The conversion would have been mediated by the activity of carbamoylase enzymes, resulting in the loss of the benzoate moiety [4]. However, carbamoylase enzyme activity is known in very few species, such as the surf clam (*Spisula solida*), which typically show a toxin profile composed of decarbamoyl toxins [14].

PST are generally characterized as hydrophilic toxins, but with GC toxins, their less polar hydroxybenzoate substituents may favour GC toxin bioaccumulation and a reduction in their elimination rate [3,8]. The aim of this study was to determine, via a recently developed and validated UHPLC-HILIC-MS/MS method [15,16], the presence of GC toxins in mussels, *Mytilus galloprovincialis,* after 5 days’ exposure to *G. catenatum* and after a 24 h elimination period, under controlled laboratory conditions.

## 2. Results and Discussion

### 2.1. Toxin Profile of Gymnodinium Catenatum Strain Used for Mussel Exposure

The most abundant PST, found in the IO-13-04 *G. catenatum* strain, as determined by HILIC-UPLC–MS/MS, were the N-sulfocarbamoyl derivatives, followed by the decarbamoyl derivatives. Small levels of carbamoyl PSP toxins were also determined, as shown in Table 1. Saxitoxin and neosaxitoxin (Neo) were not detected, with GTX3 being the most abundant carbamoyl analogue found. This profile, dominated by C toxins and decarbamoyl derivatives, is typically observed in *G. catenatum* strains isolated from the Portuguese coast [17]. In addition to classic PST, hydroxybenzoyl derivatives were detected, representing a significant part of the toxin profile of *G. catenatum.* GC1 to GC6 were here identified as previously found [6] in a different *G. catenatum* strain, isolated from the Portuguese coast, but none of the di-hydroxybenzoyl or sulfobenzoyl GC analogues indicated [4,7] were observed.

The hydroxybenzoyl derivatives appeared to be a high component of the *G. catenatum* strains, reaching similar levels to the most abundant derivatives, the C toxins. This study was also in agreement with a previous study conducted [6], showing the β-epimer forms of the N-sulfocarbamoyl and hydroxybenzoyl derivatives, which was the case for C2, GC5, GC2, and C4, as the most abundant toxin derivatives.

A culture of this *G. catenatum* strain was used to feed mussels and investigate the fate of the hydroxybenzoyl derivatives. 

### 2.2. Accumulation, Transformation, and Elimination of PST in Mussels

**Figure 2:**
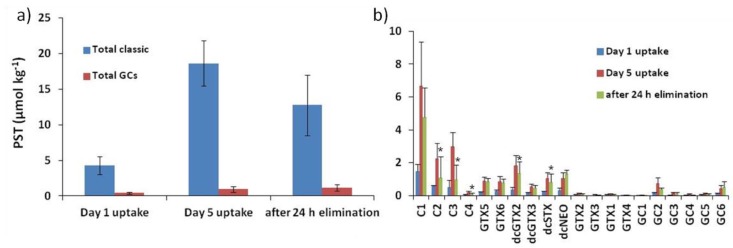
Concentration (µmol kg^−1^, mean ± SD) of paralytic shellfish poisoning toxins: (**a**) Total sums of classic PST and GC toxins; (**b**) variability of each toxin analogue, determined in mussels (*Mytilus galloprovincialis*) exposed to a toxic dinoflagellate (*Gymnodinium catenatum*) for 1 and 5 days, after 24 h of elimination. Values marked with an asterisk represent significant differences (*p* < 0.001) of toxin concentration between Day 5 uptake and 24 h of elimination.

The profile of PST determined in mussels was different to the toxin profile observed in the *Gymnodinium catenatum* strain. Although the same toxins were found in both organisms (i.e., microalgae and bivalve molluscs), the relative abundance markedly changed between the two, particularly the relative proportion of hydroxybenzoate analogues, with approximately 41% in microalgae and 9% in mussels, as shown in Figure 2A.

In mussels, the most abundant PST were the low potency N-sulfocarbamoyl derivatives, namely the C toxins, in particular the α-epimers, C1 and C3, as shown in Figure 2B. The N-sulfocarbamoyl derivatives, including the gonyautoxin 5 and 6, represented up to 71% of the toxin molar fraction of mussels, after 5 days’ exposure to *G. catenatum*. The second group of PST, in terms of abundance, were the decarbamoyl derivatives, reaching nearly 23% of the toxin profile. It is relevant to note that decarbamoyl toxins only accounted for about 4% in algae. The relative abundance of decarbamoyl toxins highly contrasted with the GC toxins that were dominant in algae and at a reduced level in mussels. The concentration of each GC toxin determined in mussels, throughout the experiment, is reported in Table 2.

Previous authors have suggested the loss of the benzoyl moiety of GC toxins would change them into their decarbamoyl toxin analogues, namely, GC3 into dcSTX, and GC1 and 2 into dcGTX2 and 3 [18]. Increasing the proportion of decarbamoyl derivatives has been described for certain species, such as the surf clam (*Spisula solida*) and the peppery furrow shell (*Scrobicularia plana*), which contain enzymes that catalyse the hydrolysis of the carboxyl bond in N-sulfocarbamoyl derivatives, leading to toxin profiles completely dominated by decarbamoyl saxitoxin derivatives [14,18,19]. Since mussels lack carbamoylase activity and levels of decarbamoyl derivatives are not as high as registered for the previously mentioned species, the increase of decarbamoyl derivatives in mussels may be related to different unknown processes that require further investigation.

After the 5-day uptake period and changing the mussels’ diet to non-toxic algae, a reduction of PST derivatives was mainly observed among the C toxins. Levels of GC toxins remained similar throughout the experiment. The carbamoyl derivatives were determined at low levels in each sampling point, and STX and Neo were not found. 

In terms of toxicity—calculated as saxitoxin equivalents—after applying the TEF (toxicity equivalence factors), as previously suggested [20], mussels reached 339 and 1359 µg STX equiv.kg^−1^, on Day 1 and Day 5 of exposure, respectively. Toxicity decreased to 1041 µg STX equiv.kg^−1^ after the 24 h elimination period. GC toxins were not taken into account despite their molar fraction of nearly 10%, due to the lack of knowledge on their toxicity potential.

## 3. Conclusions

Although the most potent PST, namely saxitoxin and neosaxitoxin, are not commonly produced by *Gymnodinium catenatum*—which may lead one to underestimate the risk of natural blooms of *G. catenatum*—this species produces a wide array of toxin derivatives, challenging environmental researchers and governmental agencies with environmental responsibilities. The hydroxybenzoate saxitoxin analogues that have been found to represent an important fraction of the toxins produced by *G. catenatum* were here found to reach nearly 10% of the toxins accumulated in mussels.

The recent move away from biological assays to chemical methods for official control of PST in shellfish, in the EU, has raised several issues, including the expression of the concentration of various compounds in an equivalent value of a single toxin. This issue has led the scientific community and competent authorities to revise the toxicity equivalency factors for PST [20,21,22,23]. However, these studies were limited to the classic PST and did not include the GC toxins. The few data available, namely, studies of affinity on rat brain sodium channels [8], suggest toxicity slightly less potent than saxitoxin. The relative abundance of GC toxins was approximately 10% in the present study, but a simple exercise of applying to GC toxins the TEF of the corresponding hydrophilic PST analogue could increase mussel PSP toxicity by 25%. Thus, it is pertinent to further investigate their toxicity potential and open a discussion about their inclusion in monitoring programs and control of PSP regulatory limits.

## 4. Materials and Methods

### 4.1. Gymnodinium Catenatum Cultivation

The *G. catenatum* strain, IO-13-04, obtained from the algae culture collection at Lisbon University (ALISU), was isolated from a bloom in Espinho, along the NW Portuguese coast, in September 2005. Cells were mass cultured in 2 L flasks, with seawater adjusted to 30‰, and enriched with a GSe medium, as done in a previous study [24], without the soil extract. Seawater and nutrient solutions were filtered (Whatman GF/C with a nominal pore size of 1.2 μm) and autoclaved to minimize contamination. The cultures were grown at 18 °C with a 12:12 L:D cycle, under fluorescent lights. Cells were harvested when cultures presented a density of approximately 2.5 × 10^6^ cells L^−1^ and concentrated, using 10 μm mesh sieve.

### 4.2. Mussels Exposure to Toxic Dinoflagellates

Immature mussels, *Mytilus galloprovincialis* (53.78 ± 6.22 mm), were harvested in Aveiro Lagoon, along the NW Portuguese coast, in July 2016, when blooms of *G. catenatum* were not occurring. Upon collection, mussels were immediately shipped to IPMA facilities (Lisbon, Portugal) in a thermally isolated container. Mussels were cleaned of macro-algae, barnacles, or any other epibiota, and placed in 150 L tank system, subdivided in 3 replicates, as described in a previous study [25]. During acclimation to laboratory conditions, mussels were fed with 100,000 cells per day, per animal, of the non-toxic and freeze-dried *Tetraselmis* sp. diet (Necton, Olhão, Portugal). The mussels were then fed for 5 days with toxic *G. catenatum* to approximately 91,000 cells per day, per animal. After the 5 days of exposure to the toxic diet, mussels were fed again with 100,000 cells of *Tetraselmis* sp. per animal, for 1 day, in order to assess the elimination of toxins. Two mussels exposed to *G. catenatum* were collected in triplicate for toxin analysis on Days 1 and 5, corresponding to the uptake period, and on Day 6, corresponding to the 24 h elimination period. Five days was considered as the mean time of a *G. catenatum* bloom peak [26,27] and 24 h was the period selected to observe the elimination of approximately 60% of the toxicity, as reported earlier [25,28].

### 4.3. Determination of PST

#### 4.3.1. Reagents

All reagents used for toxin extraction and analysis were of analytical grade or higher. Acetic acid glacial (100%, p.a.), methanol (>99.8%, p.a.), and acetonitrile (analytical grade) were obtained from Sigma-Aldrich (Sintra, Portugal). Toxins standard solutions for carbamoyl, dicarbamoyl, and N-sulfocarboyl PST analogues were purchased from the Certified Reference Materials Program of the Institute for Biotoxin Metrology, National Research Council (NRC, Halifax, Canada).4.3.2. Toxin Extraction and Sample Clean-Up

Extraction of toxins from *G. catenatum* cell cultures followed a methodology previously described [17]. A total of 0.5 L of *G. catenatum* cell culture containing 31.7 × 10^3^ cells L^−1^ was filtered onto 47 mm Whatman GF/C with a nominal pore size of 1.2 mm, under low vacuum. Toxins were extracted in 4 mL of 0.05 M acetic acid and sonicated for 4 min, at a 25 W, 50% pulse duty cycle (Vibracell, Sonic & Materials, Newtown, CT, USA) in an ice bath. Cell lysis was confirmed with light microscopy. The extract was then centrifuged (3000× *g*) for 10 min, and cleaned by carbon solid-phase extraction (SPE), as previously reported [15]. A 1 mL aliquot of the acetic acid extract was transferred to a polypropylene tube and 5 µL of NH_4_OH added. The SPE procedure was performed on a Gilson Aspec XL4 SPE liquid handling robot with amorphous graphitised polymer carbon Supelco ENVI-Carb 250 mg/3 mL cartridges (P/N:57088, Sigma–Aldrich, St. Louis, MO, USA). The cartridges were conditioned with 3 mL of acetonitrile/water/acetic acid (20:80:1 *v*/*v*/*v*) with a 200 µL air push, followed by 3 mL of water/NH_4_OH (1000:1 *v*/*v*) with a 200 µL air push, eluting to waste. 400 µL of sample extracts were loaded onto the conditioned cartridges, with a 200 µL air push, and were washed with 700 µL of deionised water with a 400 µL air push, eluting to waste. PST were then eluted with 2 mL of acetonitrile/water/acetic acid (20:80:1 *v*/*v*/*v*) with a 400 µL air push, into a labelled 5 mL polypropylene test tube. The eluent was mixed and then diluted by transferring 100 µL to a polypropylene autosampler vial and adding 300 µL of acetonitrile before analysis.

A portion (3 g) of shellfish whole body soft tissue homogenate was double extracted with 1% acetic acid solution (the homogenate was first extracted with heating), and the extracts were cleaned as described above.

#### 4.3.2. Determination of PST by HILIC-UPLC–MS/MS

An Agilent (Manchester, UK) 6495B tandem quadrupole mass spectrometer (MS/MS) coupled with an Agilent 1290 Infinity II UHPLC with a hydrophilic interaction liquid chromatography (HILIC) UHPLC column (1.7 µm, 2.1 × 150 mm Waters Acquity BEH Amide UPLC column, in conjunction with a Waters VanGuard BEH Amide guard cartridge, Waters, Manchester, UK) was used. The method was set up to detect and quantify all the classic PST carbamoyl, decarbamoyl, and N-sulfocarbamoyl analogues as described [16] and the benzoate analogues as described [6]. All other method conditions and limits of detection were as described in another study [16]. The molar concentrations of GC1, GC2, GC3, GC4, GC5, and GC6 were estimated using standards of structurally related compounds, respectively GTX2, GTX3, STX, GTX1, GTX4, and Neo. Toxicity equivalence factors used were based on those reported by the European Food Safety Autorithy—EFSA [20].

#### 4.3.3. Statistics

A one–way analysis of variance (ANOVA) was performed to test significant differences among toxins, accumulated after five days of exposure to *Gymnodinium catenatum* and 24 h feeding on non-toxic diet. Differences between means were considered significant when *p* < 0.001. Analyses were performed with Sigma Stat v3.5. 

## Figures and Tables

**Table 1:** PSP (paralytic shellfish poisoning) toxin profile of the *Gymnodinium catenatum* culture used to feed mussels.

PSP Toxins		Toxin Concentration (fmol/cell)	Molar Fraction (%)
N-sulfocarbamoyl	C1	2.0	10.7
	C2	5.6	30.1
	C3	0.2	1.1
	C4	1.4	7.3
	GTX5	0.2	1.2
	GTX6	0.2	1.2
Decarbamoyl	dcSTX	0.3	1.4
	dcNeo	0.2	1.2
	dcGTX2	0.1	0.2
	dcGTX3	0.3	1.4
Carbamoyl	GTX1	0.1	0.1
	GTX2	0.1	0.2
	GTX3	0.5	2.6
	GTX4	0.1	0.5
	STX	*<LOD*	---
	Neo	*<LOD*	---
Hydroxybenzoyl	GC1 *	0.1	0.1
	GC2 *	1.7	9.0
	GC3 *	0.4	2.0
	GC4 *	1.2	6.6
	GC5 *	3.3	17.5
	GC6 *	1.0	5.5

*<LOD* = below detection limit. * is placed after an indirect quantification.

**Table 2:** Mean concentration of hydroxybenzoate saxitoxin analogues (GC toxins) determined in mussels (*Mytilus galloprovincialis*), during uptake and elimination phases.

Toxin	GC Toxins Concentration (µmol.kg^−1^)		
	Day 1	Day 5	24 h Elimination
GC1	0.009	0.020	0.008
GC2	0.158	0.763	0.412
GC3	0.024	0.164	0.127
GC4	0.030	0.082	0.052
GC5	0.062	0.128	0.096
GC6	0.118	0.418	0.513
